# Early B-cell Factor gene association with multiple sclerosis in the Spanish population

**DOI:** 10.1186/1471-2377-5-19

**Published:** 2005-10-28

**Authors:** Alfonso Martínez, Ana Mas, Virginia de las Heras, Rafael Arroyo, Miguel Fernández-Arquero, Emilio G de la Concha, Elena Urcelay

**Affiliations:** 1 Department of Clinical Immunology, Hospital Clinico San Carlos, Madrid, Spain; 2 Department of Neurology, Hospital Clinico San Carlos, Madrid, Spain

## Abstract

**Background:**

The etiology of multiple sclerosis (MS) is at present not fully elucidated, although it is considered to result from the interaction of environmental and genetic susceptibility factors. In this work we aimed at testing the Early B-cell Factor (*EBF1*) gene as a functional and positional candidate risk factor for this neurological disease. Axonal damage is a hallmark for multiple sclerosis clinical disability and EBF plays an evolutionarily conserved role in the expression of proteins essential for axonal pathfinding. Failure of B-cell differentiation was found in EBF-deficient mice and involvement of B-lymphocytes in MS has been suggested from their presence in cerebrospinal fluid and lesions of patients.

**Methods:**

The role of the *EBF1 *gene in multiple sclerosis susceptibility was analyzed by performing a case-control study with 356 multiple sclerosis patients and 540 ethnically matched controls comparing the *EBF1 *polymorphism rs1368297 and the microsatellite *D5S2038.*

**Results:**

Significant association of an *EBF1*-intronic polymorphism (rs1368297, A vs. T: p = 0.02; OR = 1.26 and AA vs. [TA+TT]: p = 0.02; OR = 1.39) was discovered. This association was even stronger after stratification for the well-established risk factor of multiple sclerosis in the Major Histocompatibility Complex, DRB1*1501 (AA vs. [TA+TT]: p = 0.005; OR = 1.78). A trend for association in the case-control study of another *EBF1 *marker, the allele 5 of the very informative microsatellite *D5S2038*, was corroborated by Transmission Disequilibrium Test of 53 trios (p = 0.03).

**Conclusion:**

Our data support *EBF1 *gene association with MS pathogenesis in the Spanish white population. Two genetic markers within the *EBF1 *gene have been found associated with this neurological disease, indicative either of their causative role or that of some other polymorphism in linkage disequilibrium with them.

## Background

Multiple sclerosis (MS) is one of the most common neurological diseases of young adults in Europe and North America [1]. Similarly to other common complex diseases, the interplay of genome and environment as MS susceptibility factors seems to determine the final outcome. Its precise etiology is at present unknown, even though the first genetic association with the MHC was published more than thirty years ago [2]. Genomic screens support the hypothesis that susceptibility to develop MS is determined by multiple genes with small individual contributions. To decipher those combinations of genes resulting in MS is a major goal of research. Association of MS with the HLA-DRB1*1501-DQB1*0602 haplotype has been unambiguously demonstrated [3]. The diversity of the predisposition genes is evident if we consider that the major risk allele HLA-DRB1*1501 is present only in 33% of our Spanish MS patients. New susceptibility genes are therefore actively sought worldwide. In the past, candidate gene approaches have successfully revealed associations with disease susceptibility, severity or disease course.

MS has been traditionally considered an autoimmune demyelinating disorder of the central nervous system (CNS) due to autoreactive T cells on myelin proteins. However, other cells and processes have also been involved in the MS immune attack. A role for B cells in MS pathogenesis has been suggested from their presence in the cerebrospinal fluid and lesions of MS patients [4,5]. Axonal degeneration [6] has been found in early stages of the disease [7]. Axonal loss is a reliable marker of MS clinical disability [8], although the mechanism underlying axonal damage in MS remains elusive [9,10].

B cells derive from a common lymphoid progenitor, itself derived from a multipotent bone marrow progenitor. The development of a B lymphocyte comprises multiple stages with sequential expression of genes participating in immunoglobulin gene rearrangements and signaling. B cell development depends on a number of transcription factors [11] including early B cell factor (EBF), as shown for the dramatic phenotype of EBF-deficient mice [12]. B cell differentiation to plasma cell in secondary lymphoid organs is an exquisitely regulated process requiring EBF inhibition [13] and a full recapitulation of this B cell final differentiation has been recently described in the CSF [14].

EBF [15] belongs to a family of proteins present in the animal kingdom, the Collier/Olf1/EBF proteins, and it is also expressed in neural cells of different origins. EBF plays an evolutionarily conserved role in the expression of proteins essential for axonal pathfinding and neuronal differentiation in both sensory and motor neurons [16]. In addition to the action of the EBF protein in embryonic neural development, it is expressed in the adult nervous system too [17]. Furthermore, EBF binding activates the Herpes Simplex Virus Type I *ICP0 *(Infected Cell Protein 0) gene promoter, important for productive infection and reactivation from latency [18]. This virus has been related to MS [19,20].

All this evidence prompted us to determine whether the *EBF1 *gene (coding for the first member of this family cloned in humans), located at chromosome 5q has any role in MS pathogenesis. Unfortunately, no description of functional *EBF1 *polymorphisms exists in the literature, and therefore two markers were selected based on strictly genetic parameters. The first is the highly polymorphic D5S2038 microsatellite mapping to the *EBF1 *gene and the second an intronic single nucleotide polymorphism (rs1368297). The present work shows association of these markers with MS in the Spanish cohorts tested. Most probably MS is consequence of the interaction of a limited number of genetic and environmental risk factors in a patient, which may vary from those present in other patients.

## Methods

### Patients and controls

Three hundred and fifty six consecutively recruited MS patients from a single center and 540 healthy controls, mainly blood donors and staff, were included in a case-control study approved by the Hospital Ethical committee. The MS diagnosis was established based on the Poser criteria [21] and most of these patients have been described in previous studies from our group [22].

### Genotyping

D5S2038 microsatellite was amplified with annealing temperature of 56°C using the following set of primers:

Forward: 5' FAM-GTT CAA ATC TTG CCT TTG CC-3'

Reverse: 5'-GCC ATT GCT TTG TTT ATG CA-3'

Samples were subsequently denatured and run on an ABI Prism 3100 automatic sequencer (Applied Biosystems, Foster City, CA, USA). Each sample included an internal size standard in order to achieve a highly consistent measure and the results were analyzed using the GeneScan software (Applied Biosystems) and the Local Southern method.

The *EBF1 *polymorphism (rs1368297) was analyzed by TaqMan Assays-on-Demand (C___2085085_10) from Applied Biosystems, following manufacturer suggestions.

Both genetic markers conformed to Hardy-Weinberg equilibrium in the control population.

### Statistical analysis

Allele and genotype frequencies in patients and controls were compared by the χ^2 ^test; p values were considered significant at a level of < 0.05. Odds ratio (OR) and p values were calculated using a standard computer package (Epi Info v. 6.02, CDC, Atlanta, USA). The power of the study for the SNP analyzed is above 80% considering a relative risk of 1.4 and the observed allelic frequency of 0.5 at the standard significance level of 0.05.

## Results

As microsatellites are very polymorphic and informative genetic markers, we decided to select one within the *EBF1 *gene, *D5S2038*, and to study its allele distribution in our cohorts. The case-control analysis of this microsatellite in 356 MS patients and 540 healthy controls yielded the results summarized in Table 1. As shown, the only allele displaying a trend for association was D5S2038*5, while the frequency of the other ten alleles did not change significantly when patients and controls were compared. No differences were observed when different clinical forms were compared (primary progressive vs relapsing-remitting/secondary progressive). Considering these case-control results, we performed a Transmission Disequilibrium Test (TDT) with 53 trios formed by the patients and their progenitors. Distorted transmission of the D5S2038*5 was observed too (Table 2).

**Table 1:** Allele frequencies of *EBF1 *microsatellite D5S2038 in MS patients and healthy controls.

*EBF1 *D5S2038	Controls (n = 540)	MS patients (n = 356)	p
1	2	2	0.99
2	27	15	0.58
3	55	36	0.97
4	272	176	0.78
5*	246	183	0.08
6	119	81	0.80
7	79	47	0.54
8	146	94	0.83
9	8	7	0.57
10	7	4	1.00
11	3	0	0.28

* p = 0.08; OR (95%CI) = 1.26 (0.96–1.67)

**Table 2:** Transmission Disequilibrium Test (TDT) of *EBF1 *microsatellite D5S2038 in trios (MS patient and progenitors).

*EBF1 *D5S2038	Transmitted	Non transmitted	p
3	5	9	0.91
4	23	11	0.44
5	28	15	0.03
6	3	8	0.96
7	3	7	0.94
8	11	10	0.50
9	0	2	1.00
10	1	1	0.75

We pursued to check another polymorphism within the *EBF1 *gene in order to provide more evidence in support of the association of the gene with MS. Data corresponding to the allele and genotype frequencies of the intronic SNP rs1368297 are found in Table 3. Allele A was significantly increased in the diseased population and, under a recessive inheritance model, the AA homozygous genotype also conferred predisposition to MS. Moreover, the ratio of risk: non-risk homozygous genotypes was significantly higher among multiple sclerosis patients (AA: TT: p = 0.03; OR [95%CI] = 1.56 [1.03–2.37]).

**Table 3:** Allele and genotype frequencies of the *EBF1 *polymorphism in multiple sclerosis patients and controls.

EBF rs1368297	AA	AT	TT	A	T
MS patients	125	168	58	418	284
(n = 351)	35.6%	47.8%	16.5%	59.5%	40.5%
Controls	149	267	108	565	483
(n = 524)	28.4%	50.9%	20.6%	53.9%	46.1%

AA vs. (AT+TT): p = 0.02; OR (95% CI) = 1.39 (1.03–1.88).

A vs. T: p = 0.02; OR (95% CI) = 1.26 (1.03–1.53).

The MS cohort was stratified for the well-known MHC susceptibility factor DRB1*1501 and the difference between DRB1*1501^+ ^patients and healthy controls was even stronger than in the unconditioned analysis (Table 4).

**Table 4:** Genotype frequencies of the *EBF1 *polymorphism in HLA-DRB1*1501 positive and negative multiple sclerosis patients.

EBF rs1368297	AA	AT	TT	A	T
DRB1*1501^+ ^MS patients*	51	53	19	155	91
(n = 123)	41.5%	43.1%	15.4%	63%	37%
DRB1*1501^- ^MS patients	74	115	39	263	193
(n = 228)	32.5%	50.4%	17.1%	57.7%	42.3%
Controls	149	267	108	565	483
(n = 524)	28.4%	50.9%	20.6%	53.9%	46.1%

* AA vs. (AT+TT): p = 0.005; OR (95% CI) = 1.78 (1.16–2.73)

A vs. T: p = 0.01; OR (95% CI) = 1.46 (1.08–1.96)

Finally, when simultaneous carriage of both susceptibility alleles, D5S2038*5 and *EBF *SNP*A, was compared between MS patients and controls an increment was observed within the diseased cohort (p = 0.03; OR [95%CI] = 1.38 [1.03–1.84]).

## Discussion

Information from genomic screens proposed the 5q chromosomal region as linked to MS [23,24]. Additionally, a recent report compared chromosomal regions, quantitative trait loci (QTLs), of MS patients and of EAE animal models and, by analysis of sequence similarities, defined consensus genes potentially conferring susceptibility to MS [25]. Among them, the *EBF1 *gene in chromosome 5q34 was cited, providing positional evidence of the role of this gene in MS predisposition.

Moreover, the simultaneous measurement of thousands of genes upregulated by EBF through microarray technology allowed the detection of 3.5-fold increase in the expression of interleukin 6 (IL-6) and of the microtubule associated protein tau listed among the top twelve most abundant transcripts [26]. IL-6 has been detected in MS brain and its expression elevated in cerebrospinal fluid of patients [27,28]. IL-6 knockout mice showed resistance to induced EAE, too [29]. The physiological function of tau is to bind to and stabilize microtubules [30] and it is involved in regulation of axonal transport [31]. Tau protein concentration has been found repeatedly increased in cerebrospinal fluid of MS patients [32]. Also morphological examination demonstrated accumulation of amorphous deposits of abnormally phosphorylated tau in the cell body and axons of neurons within demyelinating plaques in EAE [33]. Axonopathy has been involved recently in early stages of the pathogenesis of another neurological disease, Alzheimer's disease [34]. Aberrant accumulation of proteins may be crucial to the impairment of axonal transport.

Our results evidence association of the *EBF1 *gene with MS. There are no functional studies of these gene polymorphisms, although it was cloned more than a decade ago. However, several reports showed transcriptional regulatory elements located in intronic regions of different genes [35-38]. In fact, the susceptibility allele of this EBF-intronic polymorphism allows the putative binding of an AP-1 transcription factor and this binding site is disrupted in the presence of the T allele (as predicted by TFSEARCH ver 1.3). Functional studies of *EBF1 *will aid in clarifying the role of this gene in MS pathogenesis. Nonetheless, the polymorphisms studied in this work act as genetic markers, which could potentially be the etiologic variants or be in linkage disequilibrium with them.

The EBF prototypical regulatory activity in B lymphocyte differentiation alone justifies the functional involvement of the *EBF1 *gene in an autoimmune disease as MS. Increasing evidence supports the role in MS disease course of IgM antibodies [39] produced by CD5^+ ^B-lymphocytes, that are elevated in CSF of patients with aggressive forms of MS [40]. These natural IgM antibodies recognize myelin antigens and are strong complement activators [41]. Both, antibodies and complement, have been shown to contribute to MS disability through demyelination and axonal damage [42]. IgG antibodies with hypermutated V regions have been also described [43]. Moreover, *EBF1 *is a potent modulator of adipogenesis [44] and the IgM bands in cerebrospinal fluid of MS patients were directed against myelin lipids [40].

## Conclusion

Our data suggest that the *EBF1 *gene involved in B-cell development, adipogenesis and axonal damage play a causative role in MS. Many mechanistic ties between axonal damage, tau pathology, intrathecal B1 subpopulation responsible for IgM secretion, conventional B cells, and the *EBF1 *gene role in MS susceptibility could be thought up. Confirmation in an independent cohort would substantiate our hypothesis about the implications of this gene in MS. Further understanding of the MS pathogenesis will help in the selection of therapeutic targets and characterization of the specific susceptibility genetic pattern in an individual will aid in a better diagnosis and ultimately in achievement of a personalized therapy.

## List of abbreviations used

Early B-cell Factor gene (*EBF1*).

Multiple sclerosis (MS).

Major Histocompatibility Complex (MHC).

Human leukocyte antigen (HLA).

Central nervous system (CNS).

Infected Cell Protein 0 (ICP0).

Transmission Disequilibrium Test (TDT).

Single nucleotide polymorphism (SNP).

Quantitative trait loci (QTLs).

Experimental autoimmune encephalitis (EAE).

Odds ratio (OR).

## Competing interests

The author(s) declare that they have no competing interests.

## Authors' contributions

AMartínez carried out the genotyping of some samples and participated in the statistical analysis and writing of the manuscript.

AMas carried out the genotyping of most of the patients and a great part of the controls and participated in the statistical analysis.

VdlH made the diagnosis, participated in the recollection of samples and collaborated in the statistical analysis.

RA made the diagnosis, participated in the recollection of samples and collaborated in the statistical analysis.

MFA participated in the coordination of the study and helped to collect the DNA samples and to interpret the data.

EgdlC participated in the design and coordination of the study and critically revised the article.

EU conceived of the study and participated in the statistical analysis and drafted the major part of the manuscript.

All authors have read and approved the final manuscript.

## Pre-publication history

The pre-publication history for this paper can be accessed here:


